# Temporal trends in coverage, quality and equity of maternal and child health services in Rwanda, 2000–2015

**DOI:** 10.1136/bmjgh-2020-002768

**Published:** 2020-11-13

**Authors:** Celestin Hategeka, Catherine Arsenault, Margaret E Kruk

**Affiliations:** Department of Global Health and Population, Harvard TH Chan School of Public Health, Boston, Massachusetts, USA

**Keywords:** child health, maternal health, descriptive study

## Abstract

**Introduction:**

Achieving the maternal and child health (MCH)-related Sustainable Development Goals (SDG) will require equitable and effective (quality-adjusted) coverage of recommended health interventions in low- and middle-income countries. We assessed effective coverage and equity of MCH services in Rwanda in the Millennium Development Goal (MDG) era to help guide policy decisions to improve equitable health gains in the SDG era and beyond.

**Methods:**

Using four rounds of Rwanda demographic and health surveys conducted from 2000 to 2015, we identified coverage and quality indicators for five MCH services: antenatal care (ANC), delivery care, and care for child diarrhoea, suspected pneumonia and fever. We calculated crude coverage and quality in each survey and used these to estimate effective coverage. The effective coverage should be regarded as an upper bound because there were few available quality measures. We also described equity in effective coverage of these five MCH services over time across the wealth index, area of residence and maternal education using equiplots.

**Results:**

A total of 48 910 women aged 15–49 years and 33 429 children under 5 years were included across the four survey rounds. In 2015, average effective coverage was 33.2% (range 19.9%–44.2%) across all five MCH services, 30.1% (range 19.9%–40.2%) for maternal health services (average of ANC and delivery) and 35.3% (range 27.3%–44.2%) for sick child care (diarrhoea, pneumonia and fever). This is in contrast to crude coverage which averaged 56.5% (range 43.6%–90.7%) across all five MCH services, 67.3% (range 43.9%–90.7%) for maternal health services and 49.2% (range 43.6%–53.9%) for sick child care. Between 2010 and 2015 effective coverage increased by 154.2% (range 127.3%–170.0%) for maternal health services and by 27.4% (range 4.2%–79.6%) for sick child care. These increases were associated with widening socioeconomic inequalities in effective coverage for maternal health services, and narrowing inequalities in effective coverage for sick child care.

**Conclusion:**

While effective coverage of common MCH services generally improved in the MDG era, it still lagged substantially behind crude coverage for the same services due to low-quality care. Overall, effective coverage of MCH services remained suboptimal and inequitable. Policies should focus on improving effective coverage of these services and reducing inequities.

**Key questions**

**What is already known?**

- Improving both coverage and quality of healthcare services—jointly expressed as effective coverage—is necessary to maximise the impact of health systems.
- Crude coverage of maternal and child health (MCH) services has improved in the Millennium Development Goal (MDG) era in Rwanda.

**What are the new findings?**

- While effective coverage of MCH services has improved in the MDG era, it still lagged considerably behind crude coverage due to low-quality care.
- Effective coverage remained inequitable across socioeconomic status, maternal education and regions; its increase was associated with widening socioeconomic inequalities for maternal health services and narrowing inequalities for sick child care.

**What do the new findings imply?**

- Closing the gap between crude and effective coverage will require improving quality for all MCH services included in this study. Efforts also remain to be made to increase antenatal care coverage and care seeking for child diarrhoea, pneumonia and fever.
- Given the magnitude of the quality challenges identified, structural rather than simply incremental health system quality improvement, will be required to help bridge the gap between coverage and effective coverage of MCH services in Rwanda.
- Tracking the performance of health systems using effective coverage of health services, especially in the era of universal health coverage, can facilitate progress monitoring and inform improvement strategies; better measurement will be needed to allow appropriate tracking of health system performance.

## Introduction

Substantial progress has been made in improving maternal and child health (MCH) outcomes in low- and middle-income countries (LMICs) during the Millennium Development Goal (MDG) era.^1 2^ However, growing evidence suggests that strategies from the past that focused heavily on maximising access to healthcare services will not suffice to address current challenges faced by health systems in the Sustainable Development Goal (SDG) era and beyond.^3–5^ Increasingly, improving effective (quality-adjusted) coverage of healthcare services has been advocated as one of the approaches to achieving the health related SDG targets in LMICs.^3–12^

Rwanda—a low-income country with approximately 12 million people, located in central-eastern Africa—is one of the countries where coverage (or utilisation) of many MCH interventions has considerably increased during the MDG era.^13 14^ The current proportions of women who have at least one antenatal care (ANC) visit with a skilled provider, health facility delivery and children’s immunisation coverage are all above 90%.^13^ Similarly, Rwanda is one of the few countries globally, and the only one in the region, that has achieved MDGs 4 and 5 for reductions in under 5 and maternal mortality. While there has been important progress to celebrate, by end of the MDG era maternal and under-5 mortality remained high at approximately 210 deaths per 100 000 live births and 50 deaths per 1000 live births, respectively.^13^ Low-quality healthcare in Rwandan health facilities could be one contributor. A recent study by Kruk *et al* showed that low-quality care could be a bigger driver of excess mortality than insufficient utilisation of healthcare services in many LMICs including Rwanda.^15^ Arguably, improving utilisation of health services without ensuring good quality cannot maximise the impact of health systems.^4^

Effective coverage, a metric that integrates both coverage and quality of health services in a single measure, has infrequently been used to measure the performance of health systems in LMICs.^4 12 16–19^ Most studies on the performance of the Rwandan health system in the MDG era have focused on coverage of health services.^20 21^ Studies that examined the quality of MCH services in Rwanda have generally focused on single measures of quality or have used cross-sectional or subnational data.^4 15 22–29^ To our knowledge, no study has assessed changes in effective coverage over time in Rwanda. In this study, we use multiple, nationally representative, cross-sectional surveys to assess effective coverage of MCH services in Rwanda, equity in effective coverage and its subnational distribution over the MDG era. Our findings may help guide policy decisions to improve equitable health gains in the SDG era and beyond.

## Methods

### Data sources

We used data from four waves of the standard Rwandan Demographic and Health Survey (RDHS) to examine temporal trends in coverage, effective coverage and equity in effective coverage of MCH services in the MDG era. The four waves include RDHS 2000, RDHS 2005, RDHS 2010 and RDHS 2015. The RDHS is a population-based cross-sectional survey carried out every 5 years using a two-stage sample design to gather a nationally representative household sample.^13^ Through face-to-face interviews and standardised questionnaires, this survey collects data including on health services utilisation across the continuum of care for MCH.^13^ Each standard RDHS wave collects data on maternal health services covering a period within the preceding 5 years of the survey. The RDHS has traditionally had a high response rate—at least 98% for women across the four waves. Our population of interest was all women of reproductive age (15–49 years) who had at least one live birth within the past 5 years preceding each survey wave, and children younger than 5 years.

We also used the Rwandan administrative boundary coordinates from the Database of Global Administrative Areas (https://gadm.org/download_world.html) to map distributions of effective coverage of MCH services across Rwandan districts.

### Measures

#### Coverage indicators

Our analysis included five MCH services: ANC, delivery and care for child diarrhoea, pneumonia and fever. We estimated crude coverage as the proportion of women or children who used or sought healthcare services among those who needed healthcare services due to true or perceived needs. For maternal health, we used two coverage indicators: ANC and health facility delivery. ANC crude coverage was estimated as the proportion of women who had at least four ANC visits with a skilled health provider during their most recent pregnancy leading to a live birth. In Rwanda, a skilled health provider includes a doctor, medical assistant, a nurse or a midwife. Health facility delivery crude coverage was estimated as the proportion of the women who delivered in a health facility (a proxy for delivery assisted by a skilled birth attendant in Rwanda) during their most recent pregnancy leading to a live birth. For child health, we identified three indicators for care seeking for children who suffered from diarrhoea, pneumonia or fever. Care seeking for child diarrhoea, pneumonia and fever were estimated, respectively, as the proportion of children under 5 years who had diarrhoea, symptoms of pneumonia (a cough accompanied by short, rapid breathing and difficulty breathing as a result of a problem in the chest), or fever in the 2 weeks preceding the survey and for whom advice or treatment was sought from a facility or provider (excluding pharmacies and traditional practitioners).

#### Quality indicators

Guided by the framework of the *Lancet Global Health* Commission on High-Quality Health Systems in the SDG era, the WHO recommendations on ANC for a positive pregnancy experience, the WHO Safe Childbirth Checklist and by the Integrated Management of Childhood Illness (IMCI) guidelines, we identified available measures of quality for ANC, delivery care and sick child care in the four waves of RDHS.^4 30–32^ The quality indicators selected correspond to three domains of quality of the processes of care (care competence, system competence and positive user experience) described in the *Lancet Global Health* Commission framework (online supplemental table 1).^4^

10.1136/bmjgh-2020-002768.supp1 Supplementary data

ANC quality was measured by the proportion of women who reported receiving five basic services during consultations: blood pressure monitoring, iron supplementation, counselling about pregnancy complications and urine and blood testing among those who had at least four visits with a skilled provider. The indicator related to the quality of delivery care was whether the woman received a postpartum check-up before discharge (online supplemental table 1). Data on postpartum check-ups for women who delivered in health facilities were available only in the RDHS 2010 and 2015.^33^

We identified three quality metrics for sick child care: (1) oral rehydration therapy (ORT) for child diarrhoea, (2) antibiotics for suspected pneumonia and (3) malaria test performed for children with fever. Antibiotics for pneumonia and malaria test for children with fever were available only in the RDHS 2010 and 2015. Details on how the quality indicators were estimated are provided in online supplemental table 1.

#### Effective coverage

Drawing on effective coverage frameworks,^17 18^ we calculated effective coverage of MCH services as: Effective coverage=*Quality* of MCH services×(*Utilisation* of MCH services divided by *Need* for MCH services). As there were few available measures of quality in the RDHS, the effective coverage estimates reported in this study should be regarded as an upper bound.

#### Equity in effective coverage of MCH services

We assessed inequalities in effective coverage of MCH services from 2000 to 2015 across the wealth index, area of residence (urban, rural) and women’s education. We used the wealth index in quintiles (lowest—ie, poorest, lower, middle, higher and highest—ie, richest) as provided by the RDHS. The wealth index is estimated using a household’s ownership of a set of selected assets, housing construction materials, and types of sanitation facilities and water access and estimated using principal component analysis. Maternal education was collapsed into three levels (no education, primary, secondary and higher).

### Analytical strategy

We used descriptive statistics to summarise crude coverage and effective coverage of the five MCH services. To summarise crude coverage and effective coverage over time, we also created a composite score averaging the five services at the national level. We used four ANC visits in all analyses for ANC to capture full utilisation consistent with the WHO ANC guidelines in the MDG era. As a sensitivity test, we repeated the analysis using at least one ANC visit and provided results in online supplemental appendix. We graphed national averages for crude and effective coverage of the five MCH services from 2000 to 2015 (or 2010–2015 where data were not available). We conducted equity analyses and present findings using equiplots to show inequalities in effective coverage across wealth quintiles, maternal education and area of residence over time. As the 2000 RDHS did not include a wealth index variable, we created the index ourselves using principal component analysis based on the household asset items included in the survey.

Second, we calculated effective coverage in each of Rwanda’s 30 districts and compared changes between 2010 and 2015. Given the Rwandan administrative boundaries were last redrawn in 2006 as part of decentralisation reforms, we used the most recent administrative boundary coordinates for consistency. Specifically, we calculated average effective coverage score for MCH separately at the district level and used these scores to calculate absolute changes in effective coverage (average effective coverage in 2015 minus average effective coverage in 2010). For maternal health services, we averaged the proportions of the two effective coverage indicators (effective coverage of ANC and effective coverage of health facility delivery); and for sick child care services, we averaged three effective coverage indicators (effective coverage for care for child diarrhoea, child pneumonia and child fever). We mapped these estimates across Rwandan districts using the Database of Global Administrative Areas and QGIS V.3.10. We used Stata/SE V.13.0 to create the wealth index and all other analyses were conducted using R V.4.0.2. All analyses were adjusted for the survey design (clustering, stratification and survey weights) using a survey package in R.^34 35^

### Patient and public involvement

This research was done without patient involvement. Patients were not invited to comment on the study design and were not consulted to develop patient relevant outcomes or interpret the results. Patients were not invited to contribute to the writing or editing of this document for readability or accuracy.

## Results

### Description of the study sample

Table 1 describes the study samples across the four surveys. In total, the four surveys included 48 910 women aged 15–49 years. The proportion of women with at least one live birth in the 5 years preceding the survey decreased from 49.3% in 2000 to 44.9% in 2015. Similarly, the proportion of women with no education decreased from 29.4% in 2000 to 12.3% in 2015 (table 1). A total of 33 429 children under 5 were included across the four surveys. The proportion of children with diarrhoea, symptoms of pneumonia and fever in the 2 weeks preceding the survey decreased from 2000 to 2015 (table 1).

**Table 1:** Selection and characteristics of the study samples

Survey, year	2000	2005	2010	2015
Number of clusters or villages, n	445	462	492	492
Number of households, n	9696	10 272	12 540	12 699
**Women**				
Number of women (aged 15–49 years), n	10 421	11 321	13 671	13 497
Proportion with 1+ birth within 5 years of the survey, % (95% CI)	49.3 (48.1 to 50.6)	47.9 (46.7 to 49.1)	46.8 (45.8 to 47.9)	44.9 (43.7 to 46.0)
Maternal education, % (95% CI)				
No education	29.4 (27.8 to 31.0)	23.4 (22.2 to 24.5)	15.5 (14.6 to 16.4)	12.3 (11.6 to 13.1)
Any primary	59.9 (58.5 to 61.3)	67.1 (65.9 to 68.2)	68.3 (67.1 to 69.4)	64.3 (63.0 to 65.5)
Any secondary or higher	10.6 (9.0 to 12.2)	9.6 (8.6 to 10.5)	16.2 (14.9 to 17.4)	23.4 (21.9 to 24.7)
**Children under 5**				
Number of children, n	7922	8649	9002	7856
Children who had diarrhoea in the 2 weeks prior to the survey, % (95% CI)	17.2 (16.1 to 18.3)	14.4 (13.4 to 15.3)	13.2 (12.4 to 14.1)	11.6 (10.8 to 12.5)
Children who had symptoms suggestive of pneumonia/ARI in the 2 weeks prior to the survey, % (95% CI)	18.2 (17.0 to 19.4)	15.4 (14.2 to 16.4)	3.5 (3.1 to 3.9)	5.4 (4.7 to 5.9)
Children who had fever in the 2 weeks prior to the survey, % (95% CI)	30.3 (28.7 to 31.8)	26.2 (24.7 to 27.7)	15.8 (14.8 to 16.6)	18.7 (17.5 to 19.9)
**Crude coverage**				
Antenatal care (ANC) and facility delivery, mean (range)*	18.0% (10.4%–25.7%)	21.3% (13.4%–29.3%)	53.7% (35.5%–71.9%)	67.3% (43.9%–90.7%)
Child health services, mean (range)†	13.9% (12.4%–15.6%)	23.7% (14.3%–28.7%)	43.7% (37.3%–50.4%)	49.2% (43.6%–53.9%)
Overall MCH service coverage, mean (range)‡	15.6% (10.4%–25.7%)	22.7% (13.4%–29.3%)	47.7% (35.5%–71.9%)	56.5% (43.6%–90.7%)
**Effective coverage**				
ANC and delivery, mean (range)**§**	(–)	(–)	11.8% (8.8%–14.9%)	30.1% (19.9%–40.2%)
Child health services, mean (range)¶	(–)	(–)	27.7% (19.1%–37.7%)	35.3% (27.3%–44.2%)
Overall MCH services, mean (range)**	(–)	(–)	21.3% (8.8%–37.7%)	33.2% (19.9%–44.2%)

(–)Data not available in Rwanda Demographic and Health Survey on some aspect of quality indicators used to estimate effective coverage, for example, information on postpartum check-up and child pneumonia treatment not available before 2010.

*Average of ANC 4+ and facility delivery, and the range from minimum to maximum across estimates.

†Average of care seeking for child diarrhoea, pneumonia and fever, and the range from minimum to maximum across estimates.

‡Average of ANC 4+, facility delivery, and care seeking for child diarrhoea, pneumonia and fever, and the range from minimum to maximum across estimates.

§Average effective coverage for ANC and facility delivery, and the range from minimum to maximum across estimates.

¶Average effective coverage for child diarrhoea, pneumonia and fever, and the range from minimum to maximum across estimates.

**Average effective coverage for ANC, facility delivery, and child diarrhoea, pneumonia and fever, and the range from minimum to maximum across estimates.

ARI, acute respiratory infection; MCH, maternal and child health.

### Crude and effective coverage and equity in effective coverage of MCH services

In 2015, average crude coverage was 56.5% across all five MCH services, 67.3% for maternal health services (ANC and delivery) and 49.2% for sick child care (care for child diarrhoea, suspected pneumonia and fever) (table 1). In contrast, average effective coverage was only 33.2% across all five MCH services, 30.1% for maternal health services and 35.3% for sick child care (table 1). Between 2010 and 2015 effective coverage increased by 55.5% on average across all five MCH services (154.2% for maternal health services and 27.4% for child health services) (table 1). Online supplemental table 2 shows estimates for crude coverage, quality and effective coverage for each indicator included in this study.

### Antenatal care and delivery

**Figure 1:**
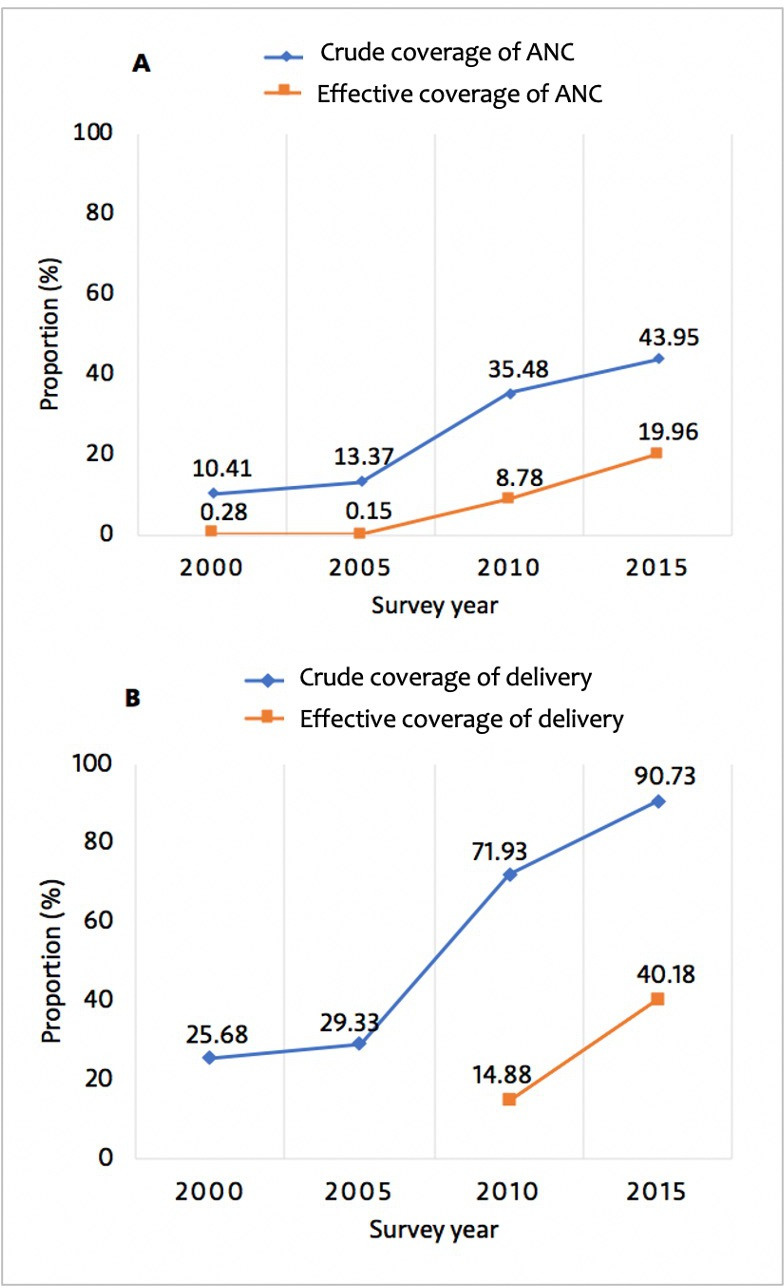
Crude coverage and effective coverage of antenatal and delivery care, 2000–2015. (A) Crude overage and effective coverage of antenatal care (ANC) (4+ visits); (B) crude coverage and effective coverage of facility delivery.

**Figure 2:**
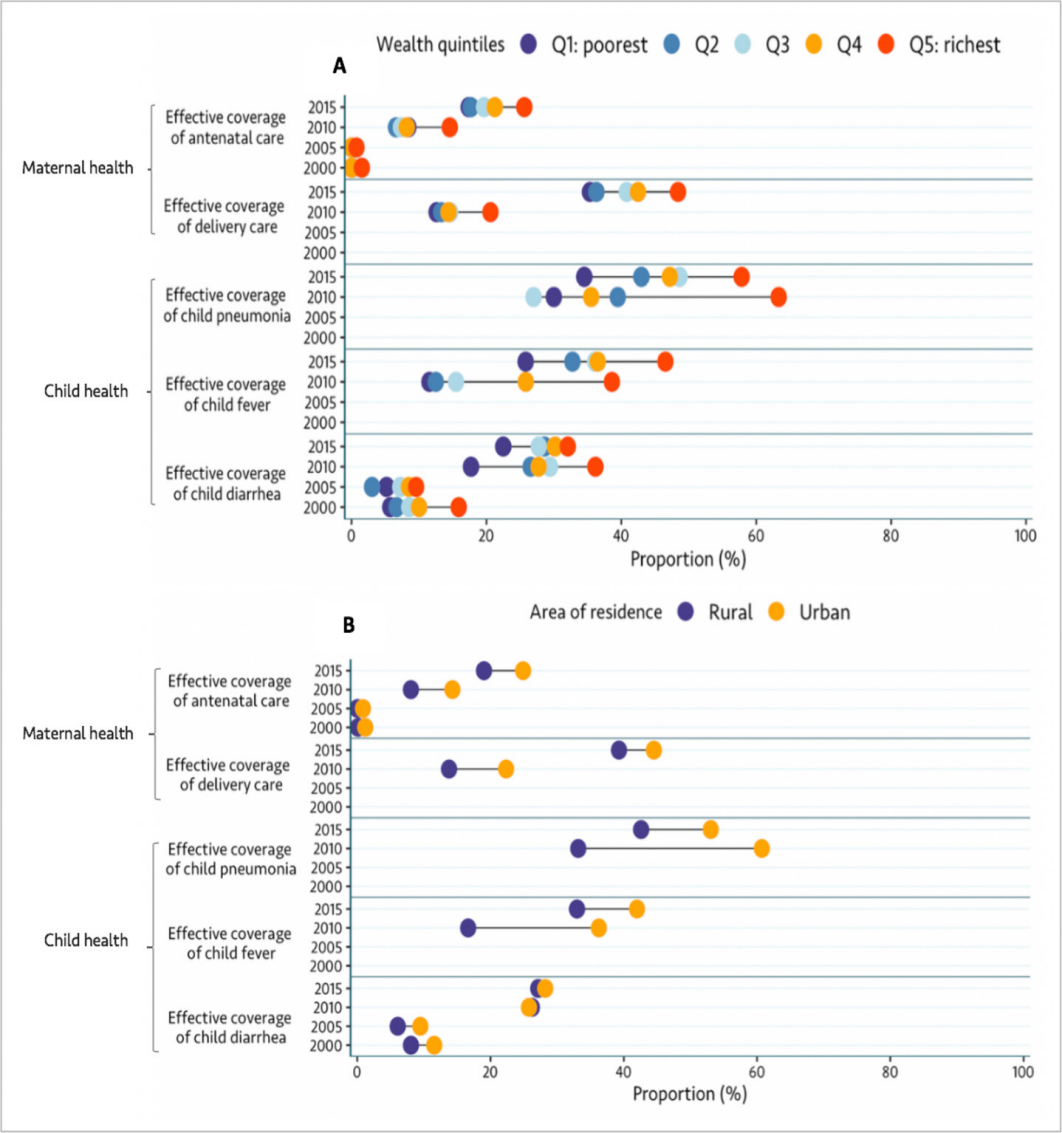
Equity in effective coverage of MCH services by wealth index (A) and area of residence (B) between 2000 and 2015. Indicators are defined in the Methods section of the manuscript and online supplemental table 1. MCH, maternal and child health.

Figure 1 shows crude coverage and effective coverage for ANC and delivery between 2000 and 2015. In 2015, effective coverage lags considerable behind crude coverage. Crude coverage was 43.9% for ANC (4+ visits) and 90.7% for facility delivery. Effective coverage was only 19.9% for ANC and 40.2% for facility delivery. Between 2010 and 2015, effective coverage increased by 127.3% for ANC and 170.0% for facility delivery. These increases were overall associated with widening socioeconomic and educational inequalities. These were largely due to widening inequalities in quality of ANC and delivery care (figure 2A and online supplemental figures 1 and 2). In contrast, urban/rural inequalities in effective coverage of delivery narrowed during the same period (figure 2B). Similarly, we observed narrowing socioeconomic, rural/urban and educational inequalities in crude coverage of ANC and delivery (online supplemental figure 3). Online supplemental figures 4 and 5A show estimates of ANC quality and receipt of the five recommended ANC components between 2000 and 2015. Looking specifically at each of the five ANC components, we noted that taking urine sample was generally less likely to be performed compared with the other ANC services (online supplemental figure 4). Online supplemental figure 5B shows the national level of quality of delivery care between 2010 and 2015.

### Sick child care

**Figure 3:**
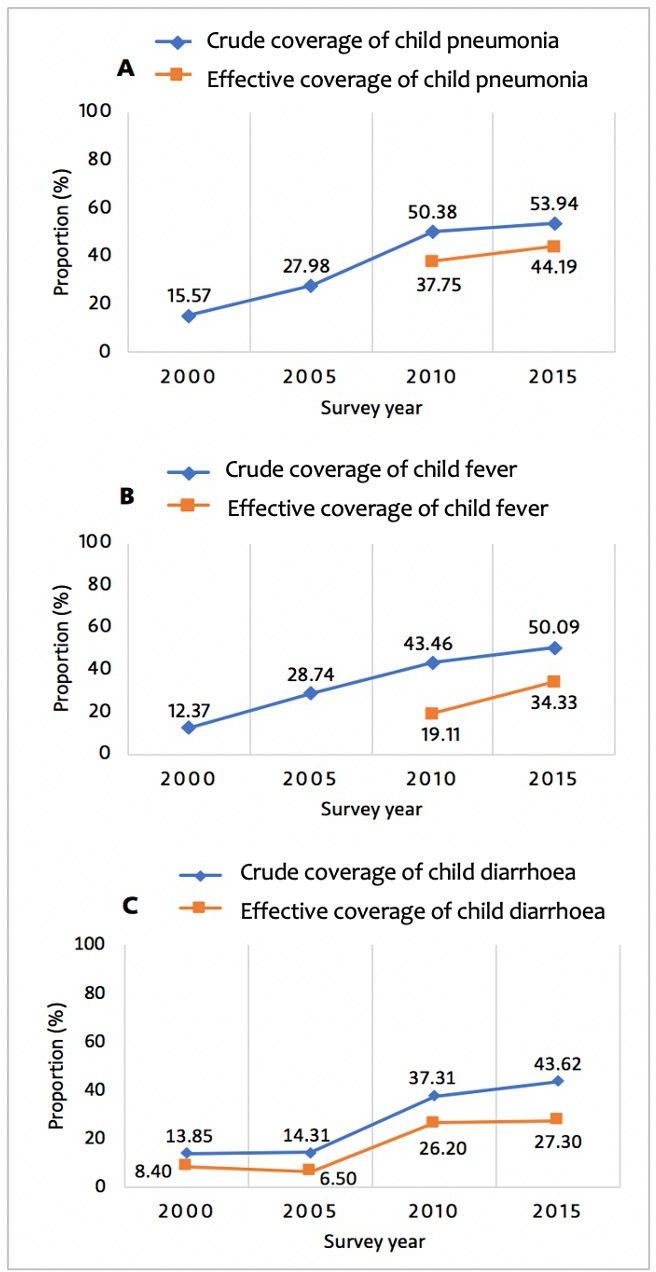
Crude coverage and effective coverage of child health services, 2000–2015. (A) Crude coverage and effective coverage of care for child pneumonia; (B) crude coverage and effective coverage of care for child fever; (C) crude coverage and effective coverage of care for child diarrhoea. Indicators are defined in the Methods section of the manuscript and online supplemental table 1.

Figure 3 shows crude and effective coverage of child diarrhoea, pneumonia and child fever between 2000 and 2015. In 2015, crude coverage (care seeking) was 53.9% for child pneumonia, 50.1% for child fever and 43.6% for child diarrhoea. In contrast, effective coverage was 44.0% for child pneumonia, 34.3% for child fever and 27.3% for child diarrhoea (figure 3). Overall, the gap between crude coverage and effective coverage was relatively smaller for child pneumonia than for child fever and diarrhoea in 2015 (figure 3 and online supplemental table 2). Between 2010 and 2015, effective coverage increased by 17.1% for child pneumonia, 79.6% for child fever and 4.2% for child diarrhoea. These increases were associated with narrowing socioeconomic, rural/urban and education inequalities for effective coverage of sick child care (figure 2 and online supplemental figure 1). Overall, inequalities in effective coverage of child diarrhoea appear smaller than those seen in effective coverage for child pneumonia and fever (figure 2 and online supplemental figure 1). Online supplemental figure 6 shows the level of quality of care for sick children between 2000 and 2015.

### Subnational distributions of effective coverage of MCH services

**Figure 4:**
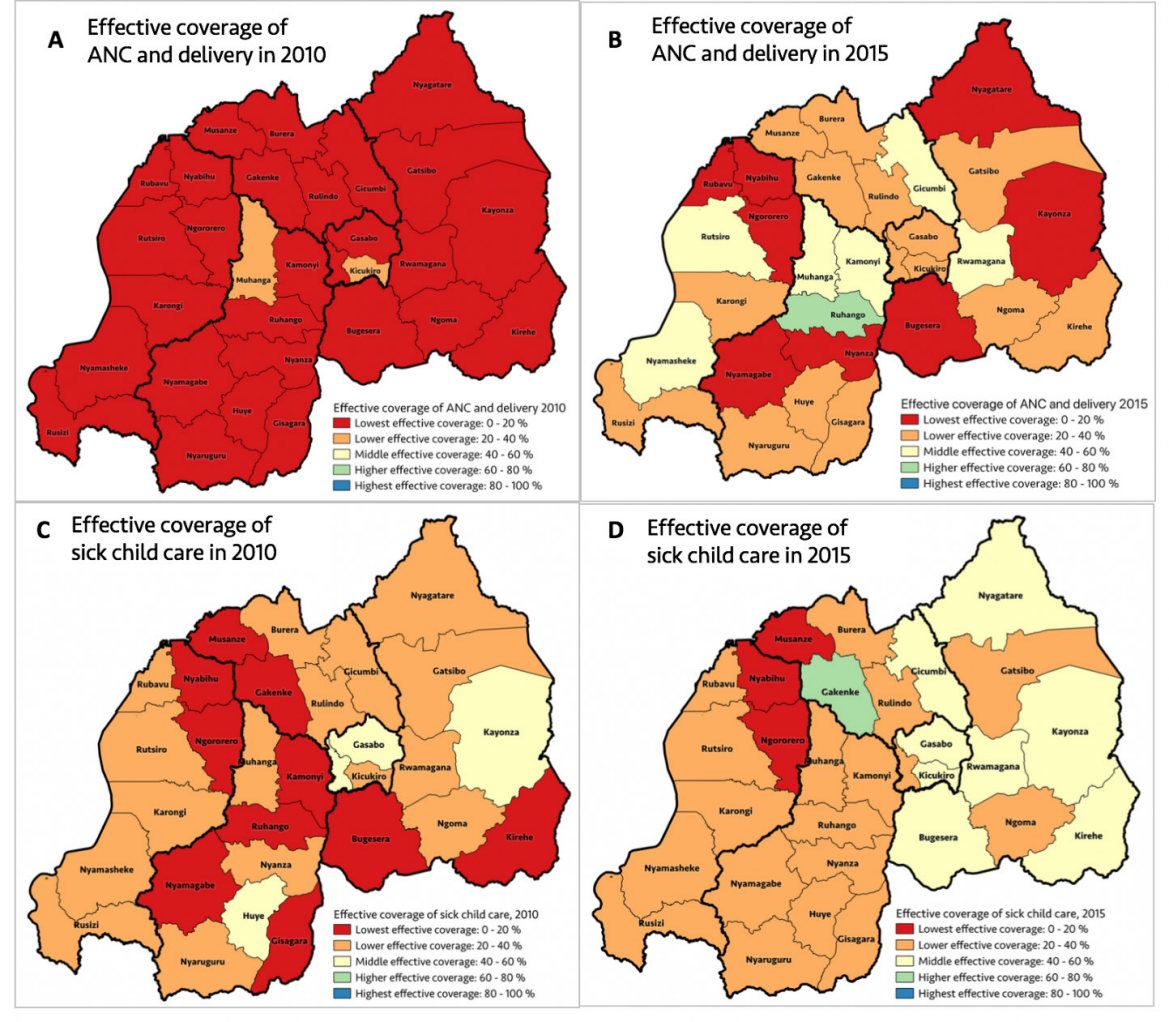
District level variations in effective coverage of MCH services, 2010–2015. (A) Effective coverage of maternal health services (average effective coverage for ANC (4+ visits) and for health facility delivery) in 2015; (B) effective coverage of maternal health services (average effective coverage for ANC (4+ visits) and for health facility delivery) in 2010; (C) effective coverage of child health services (average effective coverage for child diarrhoea, pneumonia and fever) in 2015; and (D) effective coverage of child health services (average effective coverage for child diarrhoea, pneumonia and fever) in 2010. ANC, antenatal care; MCH, maternal and child health.

Figure 4 shows variation in the average effective coverage of MCH services across 30 Rwandan districts in 2010 and 2015. Between 2010 and 2015, effective coverage of the two maternal health services improved (although unevenly) in all districts but remains low in 2015 (figure 4A, B and online supplemental figure 7A). Improvements in effective coverage of sick child care services were mixed, with some districts registering a decline from 2010 to 2015 and others showing improvement (figure 4C, D and online supplemental figure 7B).

## Discussion

In this study, we used four rounds of RDHS to estimate effective (quality-adjusted) coverage and equity of five MCH services in Rwanda during the MDG era. This study showed that while effective coverage of MCH services has improved in the MDG era and inequalities narrowed on some indicators, it still lagged considerably behind crude coverage. Moreover, by end of the MDG era, effective coverage remained largely inequitable across wealth, place of residence and maternal educational groups, highlighting a need for quality improvement efforts to ensure equitable effective coverage for all in the SDG era and beyond. Furthermore, subnational analyses illuminated considerable district-level variations in effective coverage of MCH services. Similar to our findings, studies that have evaluated health system performance for MCH services in LMICs using effective coverage have shown that effective coverage was considerably lower than crude coverage because of poor quality of care for mothers and children.^16 36 37^

Our findings show that effective coverage of MCH services improved in the MDG era as a result of overall increase in coverage and quality. This highlights health system strengthening efforts that the country has deployed and also explains at least partially why Rwanda achieved MDGs 4 and 5. Effective coverage matters more now than in the past because maternal and child mortality are lower than they were at the beginning of the MDG era. Thus, the residual deaths will be more difficult to avert without a key focus on quality (and coverage where it remains low).

For ANC, we found a substantial gap between crude and effective coverage and found that this gap generally widened between 2000 and 2015, suggesting that quality has not improved as much as utilisation. According to the most recent data, only 44% of Rwandan women received a minimum of four ANC visit consultations during pregnancy. In addition, only about one in five received a basic minimum package of five ANC components during their pregnancy including having their blood pressure monitored, urine and blood tested, receiving iron supplements, and being adequately counselled on danger signs in pregnancy and where to go in case of a complication. This echoes findings from other LMIC settings.^28^ In addition to low overall ANC quality, inequalities persisted with the wealthiest, most educated and urban women showing the greatest improvement in effective coverage of ANC. This was largely driven by wide inequalities in ANC quality consistent with previous studies.^4 27^ Our findings imply that important efforts are needed to achieve universal access to high-quality ANC in Rwanda. There will also be a need to pay attention to eliminating persistent inequalities in both ANC coverage and quality.

While health facility delivery increased considerably in Rwanda in the MDG era, with over 90% of health facility delivery coverage by 2015, effective coverage of health facility delivery still lagged behind. By 2015, only 44% of women who delivered in health facilities in Rwanda reported anyone checking on their health after giving birth before discharge from the facility. Similar to ANC, the wealthiest, most educated and urban women generally had the greatest improvement in effective coverage. Among all MCH services, the gap between crude and effective coverage was the largest for facility delivery. This is largely because crude coverage of facility delivery was highest, but quality of delivery care was very low. This suggests that, unlike ANC, optimising effective coverage of facility delivery care will largely require bolstering quality of delivery care as crude coverage is already high.

Unlike maternal health services, low effective coverage of sick child care resulted from insufficient coverage to a larger extent than low quality. Coverage of sick child care improved during the MDG era; however, it remained suboptimal (~50%) echoing findings from previous research in other LMICs.^38^ Unlike ANC and facility delivery, some episodes of fever, cough and diarrhoea may not warrant care seeking in health facilities, explaining at least partially why coverage of sick child care remains below 100%. Although inequalities in coverage of maternal health services generally narrowed in the MDG era, they appear to be persisting for sick child care. Overall, care seeking for child pneumonia and fever increased slightly faster than for child diarrhoea. Rwanda has one of the highest rates of child immunisation coverage and retention across LMICs (online supplemental figure 8).^39^ As such, applying lessons and strategies used to optimise child immunisation coverage could help to enhance care seeking for sick children in the country. Community outreach and improvements in quality of care may help to improve care seeking and public trust in the health system.^4^ Additionally, further research is necessary to understand why care seeking for child illness is overall low and its increase has been slow. Adherence to clinical practice guidelines for quality sick child care was over 60% by end of the MDG era—explaining why the gap between crude and effective coverage was relatively small—however, there still is a room for further improvements in quality consistent with findings from other studies on quality for sick child care in LMICs.^4 40^ Efforts are needed to improve health provider’s adherence to IMCI guidelines and ensure that Rwandan children who are brought to health facilities for illnesses receive all appropriate diagnostic tests, treatments and counselling needed to improve their health.

This study has several limitations. First, our quality measures are not comprehensive as they included only a limited number of recommended items that should be completed during ANC visit, delivery/postnatal care and care of sick children. For example, to ensure comparability across surveys, we used only five indicators to create an ANC quality index. Similarly, our effective coverage indicators for health facility delivery and care for pneumonia, diarrhoea and fever were created by adjusting coverage by only one indicator of quality (any postpartum check-up before discharge, antibiotics, ORT and malaria testing). These dichotomous indicators, where survey respondents answered yes or no, do not measure quality comprehensively. Other relevant indicators, such as appropriate assessment and diagnostic tests, timeliness of care and other preventive and curative treatments for each condition, were not included in the quality measures because they are not available in the RDHS.

Given these limitations, we likely overestimated effective coverage of MCH services, especially for indicators that relied on one quality measure, and underestimated inequalities in effective coverage. As such, the effective coverage should be regarded as an upper bound because there were few available quality measures and should be taken as a starting point for measuring effective coverage rather than definite measures. Clearly, better measurements, and timely data, on coverage and quality are needed. Along with data, comprehensive assessments of effective coverage and research on potential determinants are needed to generate evidence and inform improvement initiatives at the national and subnational levels in Rwanda.^4 26 29^

Current routine health information systems in Rwanda capture limited data on quality of care and health system performance.^29^ While health facility assessment surveys are important sources of data on quality of care, there is a paucity of such surveys in Rwanda. For example, the most recent Service Provision Assessment survey in Rwanda was conducted in 2006.^29 41^ Recent work on health system quality suggests that measurement of quality (from surveys and/or routine health information systems) must be improved and new measures are needed to allow effective tracking of health system performance for maternal, newborn and child health.^4 26 41 42^

Second, our analyses relied on self-reported data, which are subject to information bias including recall bias, with its effect varying by type of indicators. In particular, while invasive interventions (eg, blood tests, finger/heel stick) are more accurately self-reported, other indicators such as counselling about pregnancy complications could be more subject to recall bias.^43^ Similarly, McCarthy *et al* recently conducted a validation study of women’s reports of antenatal and postnatal care received and revealed that women generally report accurately indicators related to concrete and observable actions performed on them (eg, blood pressure check, anaemia screening and urine test) as opposed to information or advice they were offered.^44^ Moreover, research conducted in Kenya and Swaziland suggests that women generally report accurately contents of postnatal care.^45^ Furthermore, although caregiver report using DHS questions appears to be a valid measure of overall care seeking for childhood illness,^46^ validity of these reports is poor for identifying childhood pneumonia and correctly recalling antibiotic treatment.^46 47^ Indeed, poor validity of some of the indicators we analysed could have affected our effective coverage estimates especially for childhood pneumonia. However, our analysis included similar indicators across different survey years—which would theoretically make any information bias more likely systematic across different surveys—making improvement or change in crude coverage and effective coverage of MCH services less likely to be substantially affected.

## Conclusion

Effective coverage of MCH services has improved in the MDG era in Rwanda. However, important efforts are still needed to raise quality of care (and coverage where it remains low), while ensuring equity across wealth and education groups and regions of residence, in order to optimise effective coverage of the MCH services that we studied. To inform improvement strategies and ultimately maximise impact of the health system in the era of universal care coverage, effective coverage measures should be employed.^4 48^ To achieve this, better measurement and timely data are critical to enable a comprehensive assessment of effective coverage of MCH services in Rwanda. In particular, measures of care and system competence, user experience and outcomes, including health outcomes and confidence in the system should be prioritised and aligned with currently recommended best practices for antenatal and delivery care and care for child diarrhoea, pneumonia and fever.^4 26 30 32 49^ Furthermore, equity analysis is vitally important to enable identifying who gets the worst quality of care to help guide policy decisions toward equitable distribution of health gains in the SDG era and beyond. Lastly, given the size of the quality challenges identified here, structural (eg, modernised provider training, governance reforms, service delivery reforms) rather than simply incremental health system quality improvement (eg, short-term training programmes) will be required to help bridge the gap between coverage and effective coverage of MCH services in Rwanda and ultimately help reduce mortality due to low quality of care in line with national ambitions and the SDGs.^4 29^
