# Supplementary material for: Temporal trends in coverage, quality and equity of maternal and child health services in Rwanda, 2000–2015
Source: BMJ Glob Health. 2020 Nov 13;5(11):e002768. doi: 10.1136/bmjgh-2020-002768 (PMC7668303; doi:10.1136/bmjgh-2020-002768)
Supplement: Supplementary data [file bmjgh-2020-002768supp001.pdf]

BMJ Publishing Group Limited (BMJ) disclaims all liability and responsibility arising from any reliance placed on this supplemental material which has been supplied by the author(s)

Coverage, quality and equity of maternal and child health services in Rwanda: analysis of national surveys from 2000 to 2015

Celestin Hategeka MD, PhD; Catherine Arsenault PhD; Margaret E Kruk MD, MPH

Supplementary Appendix

Appendix Table 1. Quality indicators included in the Rwanda DHS mapped to the Lancet Global Health Quality Commission framework

Quality dimension	Care component	MCH service	Quality of care (Numerator)	Use of care (Denominator)
**Competent care**				
Assessment	Blood pressure, urine and blood samples taken during ANC with skilled provider	Antenatal care	Women who received all of the following at any point during their last pregnancy: blood pressure checked, urine and blood samples taken	Women who had at least four antenatal care visits with a skilled provider during their last pregnancy (doctor, nurse or midwife or medical assistant)
Assessment	Postpartum checkup for mothers in a health facility after delivery and before discharge	Postnatal care	Women who were examined or asked questions about their health before discharge	Women who delivered in a health facility
Treatment	Received antibiotics when seeking care at a facility for symptoms of pneumonia	Care of sick children	Children who received antibiotic pills, syrup or injections	Children under 5 who, in the past 2 weeks, have suffered from symptoms consistent with pneumonia (a cough accompanied by short, rapid breathing and difficulty breathing as a result of a problem in the chest) and were taken to a medical facility for treatment (including public sector and medical private sector facilities, except for pharmacies)
Assessment	Tested for malaria	Care of sick children	Children who had blood taken from finger or heel for testing	Children under 5 who had fever in the past 2 weeks and were taken to medical facility for treatment (including public sector and medical private sector facilities, except for pharmacies)
Treatment	Received oral rehydration therapy when seeking care at a facility for diarrhea	Care of sick children	Children who received oral rehydration therapy (from oral rehydration salts (ORS), pre-packaged ORS liquid or other homemade fluids)	Children under 5 who had diarrhea in past 2 weeks and were taken to a medical facility for treatment (including public sector and medical private sector facilities, except for pharmacies)
**Competent systems**				
Prevention/treatment	Iron supplementation	Antenatal care	Women who were given or bought iron supplements	Women who had at least one antenatal care visit with a skilled provider during their last pregnancy (doctor, nurse or midwife or medical assistant)
Timely care	Woman was checked within 1 hour of delivery after giving birth in a facility	Postnatal care	Women who were examined or asked questions about their health within one hour of delivery	Women who delivered in a health facility

Continuity	Received the third dose for DTP by one year of age, among those who had received the 1st dose	Children's vaccination	Children who had all three doses of the diphtheria, tetanus, pertussis vaccine by one year of age	Children who received at least one dose of the DTP vaccine
**Positive user experience** (clear communication)				
Counseling	Told about danger signs or where to go in case of complications during ANC with skilled provider	Antenatal care	Women told about potential danger signs to look for during pregnancy or where to go in case of complications	Women who had at least one antenatal care visit with a skilled provider during their last pregnancy (doctor, nurse or midwife or medical assistant)

Appendix Table 2. Coverage, quality, and effective coverage of maternal and child health services

Survey year	2000	2005	2010	2015
**Coverage, % (95% CI)**				
Antenatal and delivery care				
• ANC-any	92.8% (91.9, 93.7)	94.6% (93.7, 95.4)	98.2% (97.8, 98.5)	99.2% (98.9, 99.4)
• ANC-four visits	10.4% (9.2, 11.5)	13.4% (12.1, 14.5)	35.5% (33.9, 37.0)	43.9% (42.2, 45.6)
• Delivery care	25.7% (23.2, 28.1)	29.3% (27.4, 31.2)	71.9% (70.3, 73.5)	90.7% (89.6, 91.8)
Under-5 care seeking				
• Pneumonia (ARI)	15.6% (13.3, 17.8)	27.9% (25.2, 30.7)	50.4% (43.4, 57.3)	53.9% (49.2, 58.7)
• Fever	12.4% (10.4, 14.4)	28.7% (26.3, 31.1)	43.5% (40.4, 46.5)	50.1% (47.0, 53.1)
• Diarrhea	13.8% (11.6, 16.1)	14.3% (12.0, 16.6)	37.3% (34.2, 40.4)	43.6% (40.1, 47.2)
**Quality, % (95% CI)**				
Antenatal and delivery care				
• ANC-any	0.3% (0.14, 0.50)	0.4% (0.28, 0.65)	19.2% (17.8, 20.6)	39.4% (37.6, 41.2)
• ANC-four visits	2.7% (1.2, 4.2)	1.1% (0.4, 1.8)	24.9% (22.6, 27.2)	45.5% (43.2, 47.9)
• Delivery care	(-)	(-)	20.68% (19.4, 21.9)	44.3% (42.5, 45.9)
Sick-child care				
• Child pneumonia	(-)	(-)	74.9% (67.7, 82.1)	81.9% (76.7, 87.2)
• Child Fever	(-)	(-)	43.9% (39.5, 48.5)	68.5% (64.6, 72.5)
• Child diarrhea	60.9% (53.2, 68.7)	45.6% (37.3, 53.8)	70.1% (65.6 74.5)	62.6% (57.3, 67.8)
**Effective coverage, % (95% CI)**				
Antenatal and delivery care				
• ANC-any	0.3% (0.13, 0.46)	0.4% (0.26, 0.62)	18.9% (17.5, 20.2)	39.1% (37.3, 40.8)
• ANC-four visits	0.3% (0.11, 0.44)	0.2% (0.06, 0.24)	8.8% (7.8, 9.7)	20.0% (18.6, 21.3)
• Delivery care	(-)	(-)	14.8% (13.9, 15.8)	40.2% (38.5, 41.8)

Sick-child care				
• Child pneumonia	(-)	(-)	37.6% (31.4, 43.8)	44.0% (38.9, 48.9)
• Child Fever	(-)	(-)	19.1% (16.5, 21.6)	34.3% (31.3, 37.3)
• Child diarrhea	8.4% (6.7, 10.1)	6.5% (5.0, 8.0)	26.2% (23.4, 28.9)	27.3% (24.2, 30.4)
**Crude-effective coverage gap *, % (95% CI)**				
Antenatal and delivery care				
• ANC-any	92.4% (91.5, 93.3)	94.1% (93.3, 94.9)	79.3% (77.9, 80.6)	60.1% (58.3, 61.8)
• ANC-four visits	9.9% (8.8, 11.0)	13.1% (11.9, 14.3)	26.5% (25.1 27.9)	23.9% (22.5, 25.2)
• Delivery care	(-)	(-)	57.0% (55.4, 58.6)	50.5% (48.9, 52.2)
Sick-child care				
• Child pneumonia	(-)	(-)	12.6% (8.5, 16.6)	9.7% (6.8, 12.6)
• Child Fever	(-)	(-)	24.3% (21.8, 26.6)	15.8% (13.6, 17.8)
• Child diarrhea	5.4% (3.9, 6.8)	7.7% (6.0, 9.5)	11.2% (9.3, 13.0)	16.3% (13.6, 19.0)

CI, confidence interval; ANC, antenatal care. *Crude-effective coverage gap refers to absolute difference between crude coverage and effective coverage for each indicator across time and at the national level.

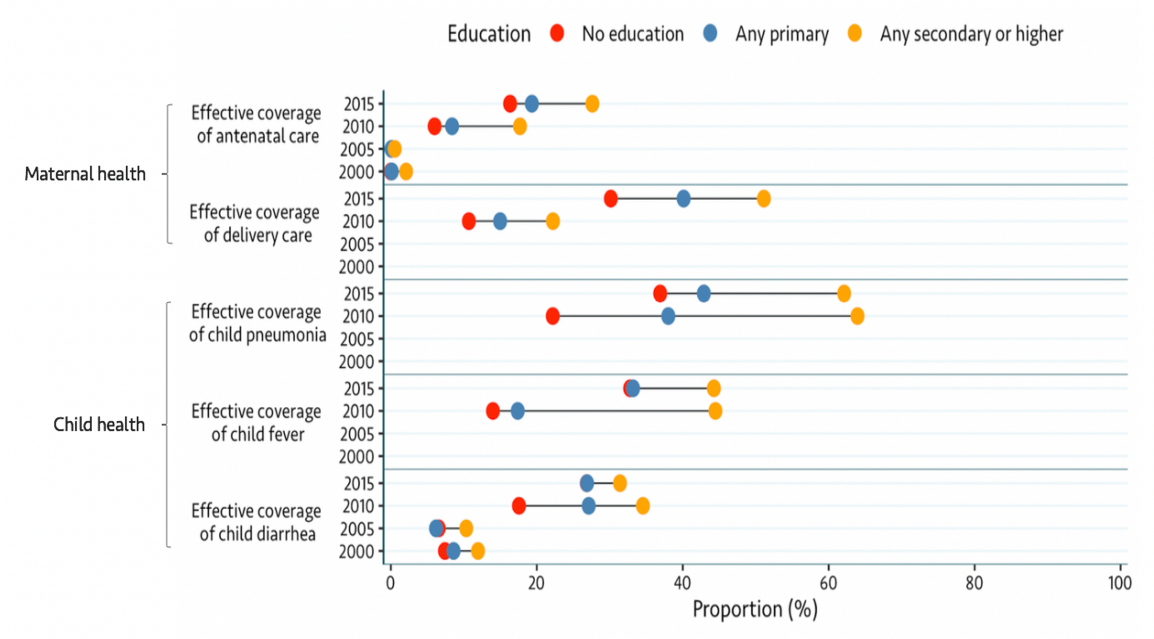

Appendix Figure 1. Equity in effective coverage of MCH services by maternal education between 2000 and 2015. Indicators are defined in Appendix Table 1.

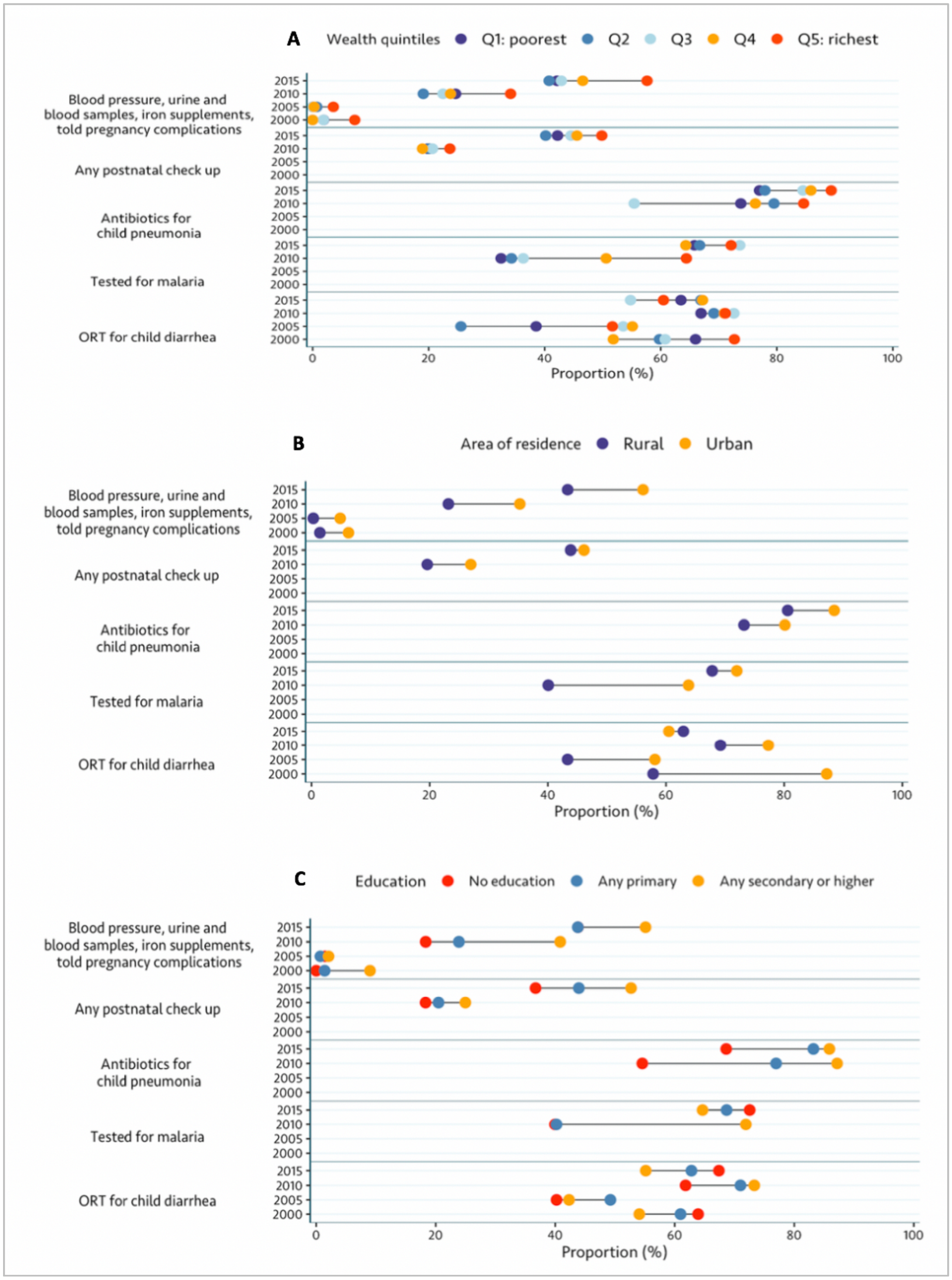

Appendix Figure 2. Equity in quality of selected MCH services by wealth index (A), area of residence (B) and maternal education (C) between 2000 and 2015.

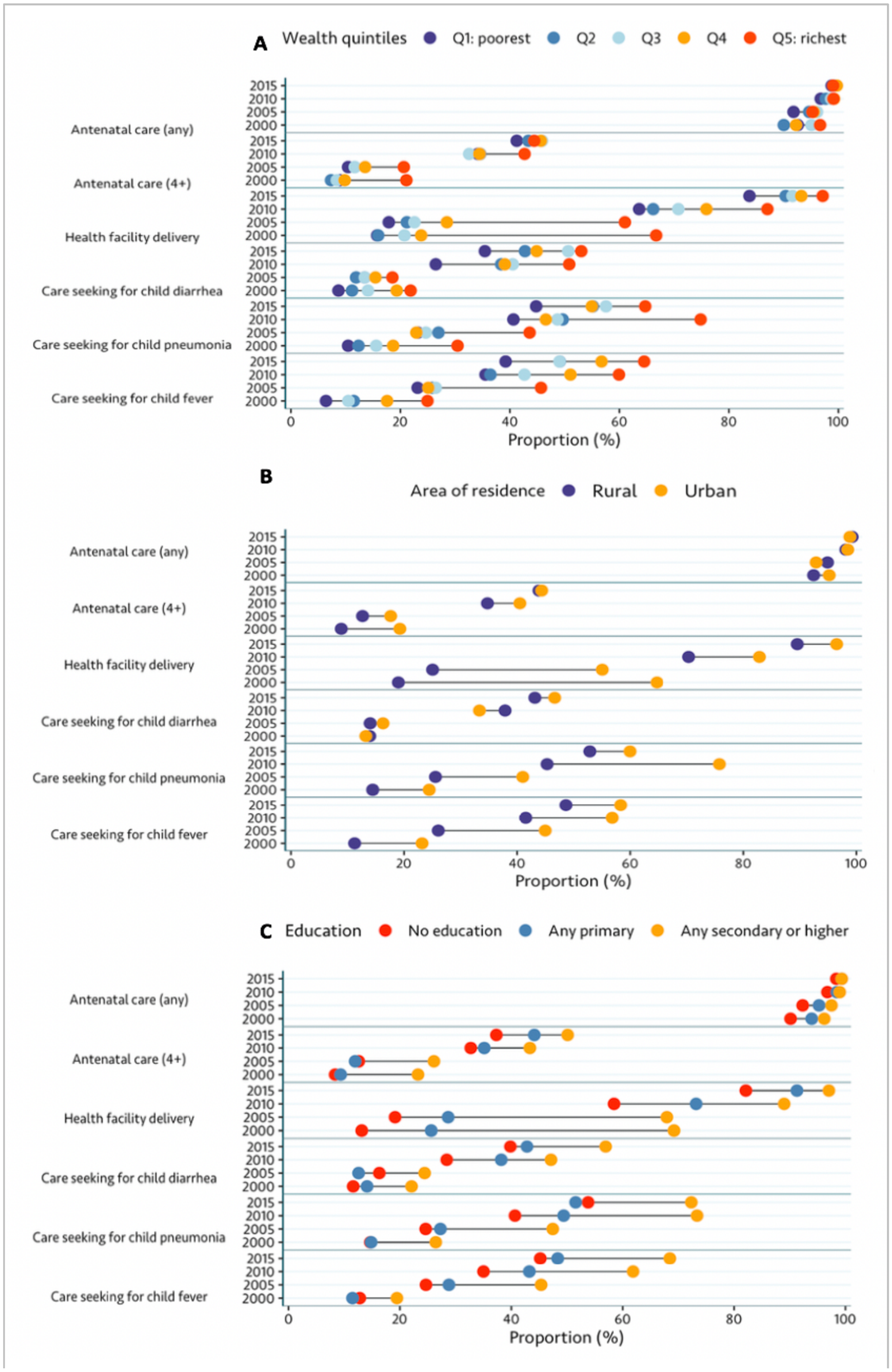

Appendix Figure 3. Equity in crude coverage of selected MCH services by wealth index (A), area of residence (B) and maternal education (C) between 2000 and 2015.

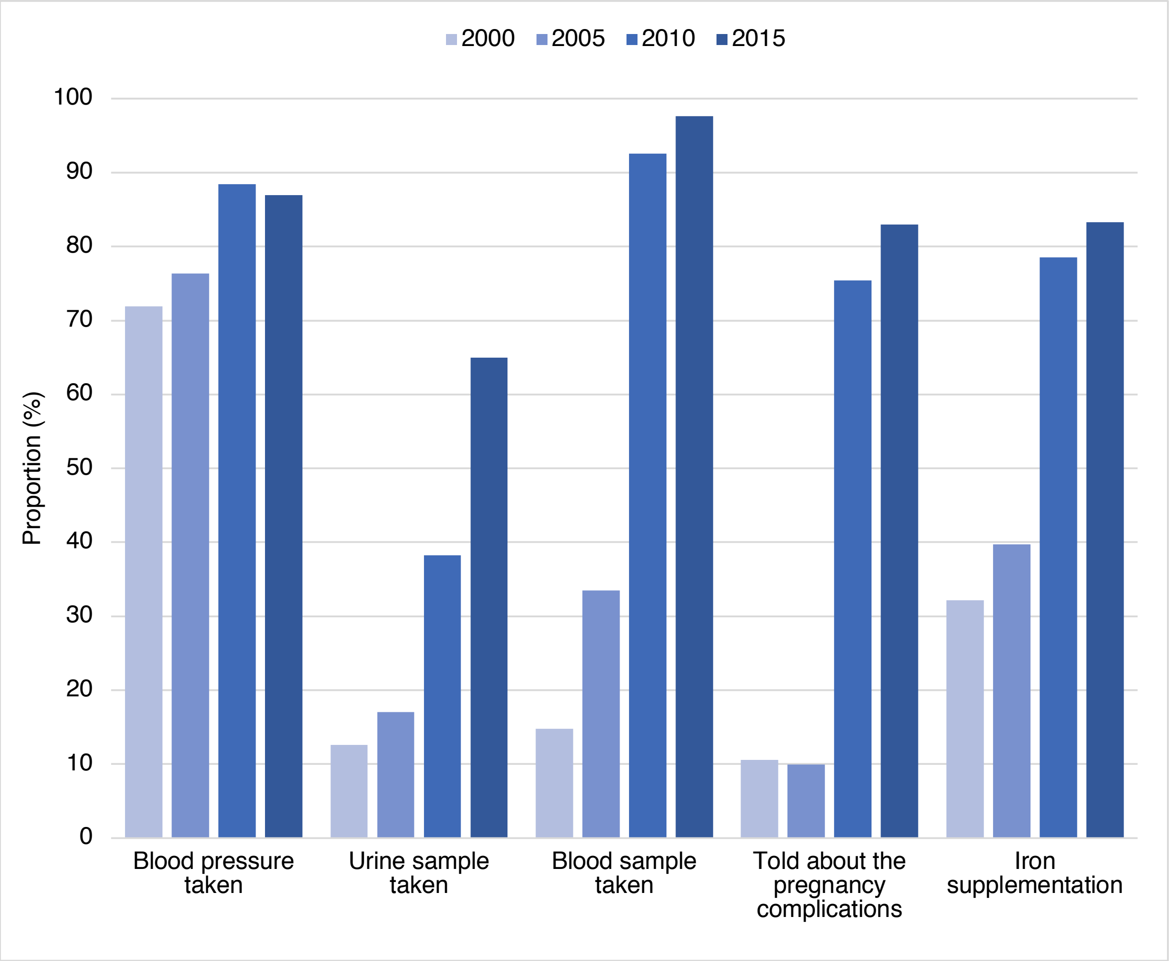

Appendix Figure 4. Content of antenatal care from 2000 to 2015. These five ANC services were used to create a binary indicator for quality of ANC referring to the proportion of women who report blood pressure monitoring, iron supplementation, counseling about pregnancy complications, urine and blood testing at any point during pregnancy among those who had at least four ANC visits with a skilled provider.

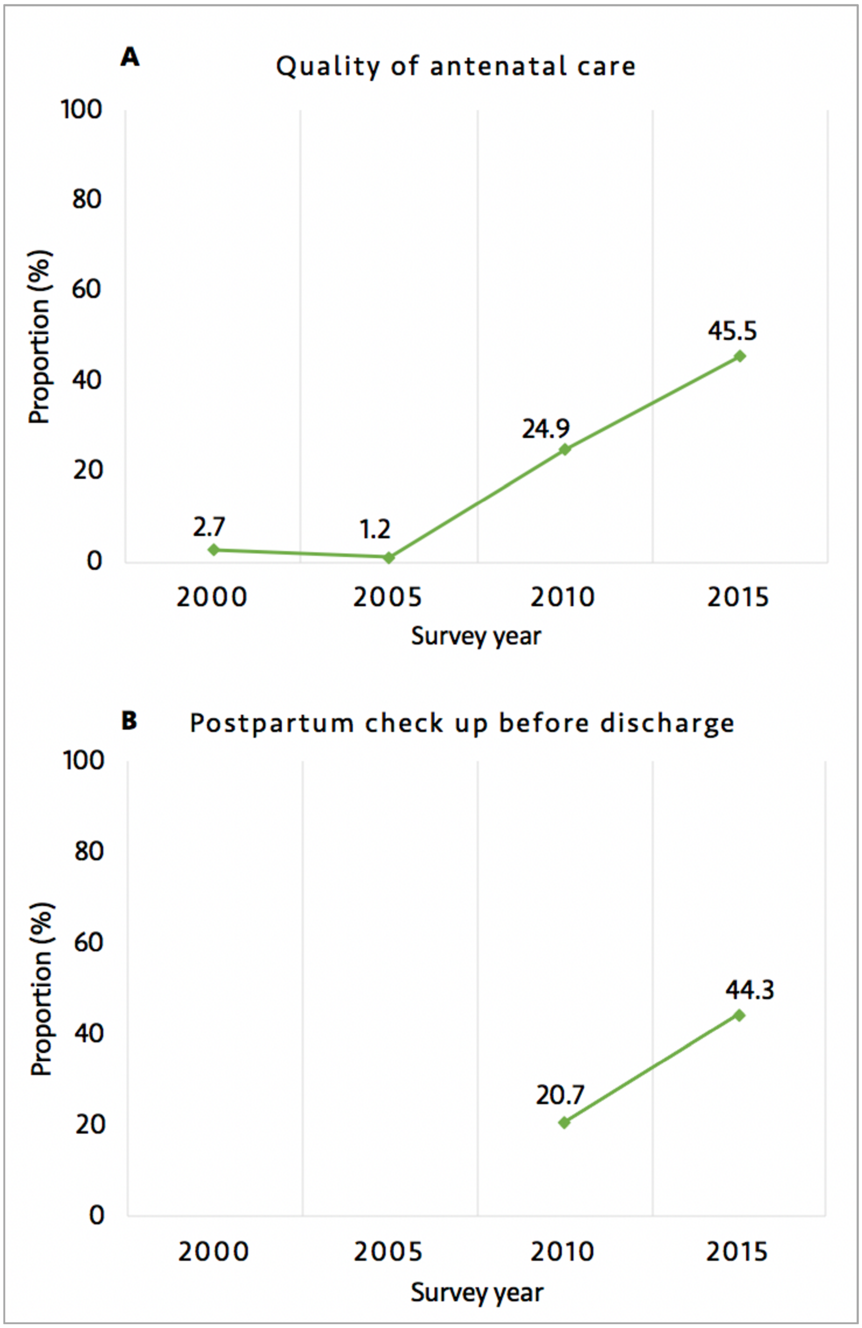

Appendix Figure 5. Quality of selected maternal health services from 2000 to 2015. Quality of antenatal care refers to the proportion of women who report blood pressure monitoring, iron supplementation, counseling about pregnancy complications, urine and blood testing at any point during pregnancy among those who had at least four ANC visits with a skilled provider.

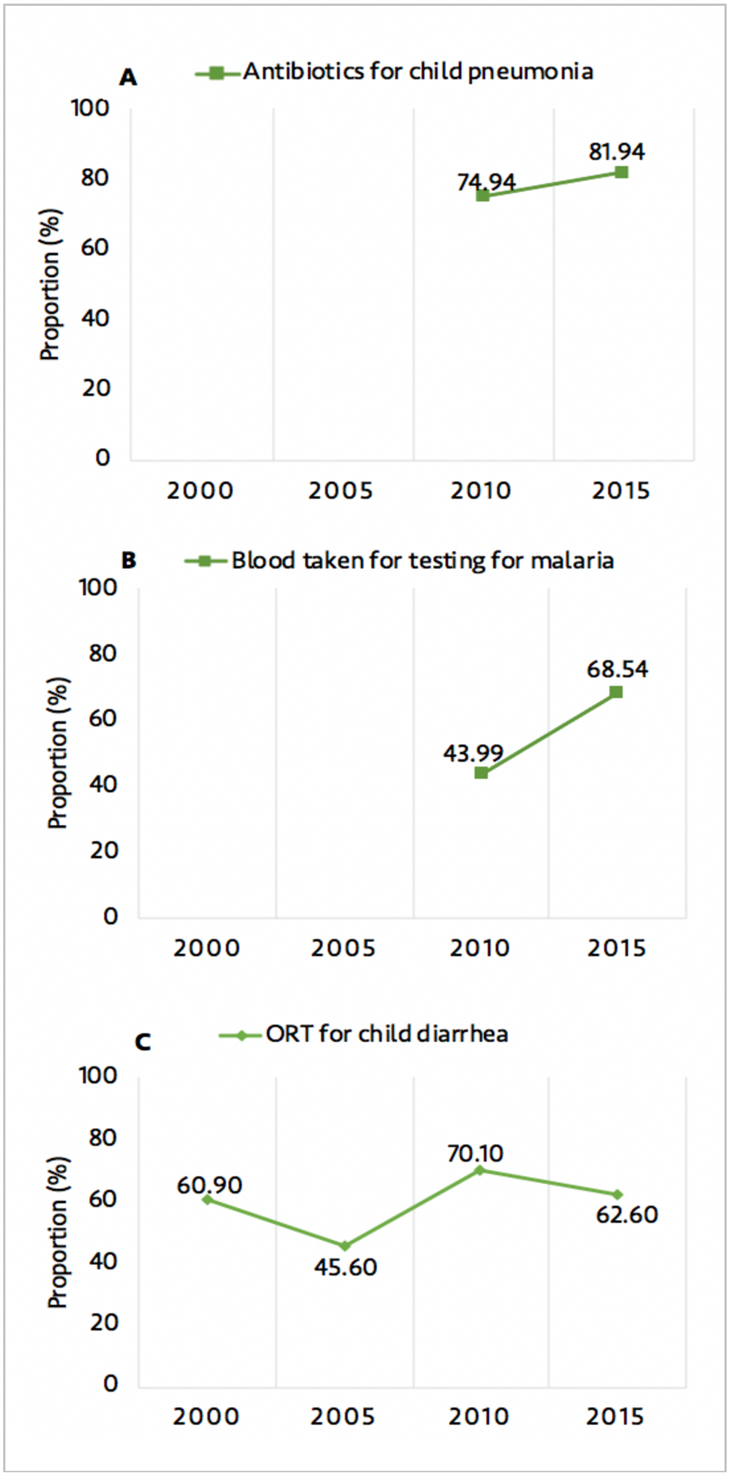

Appendix Figure 6. Quality of selected sick-child care from 2000 to 2015. ORT, oral rehydration therapy

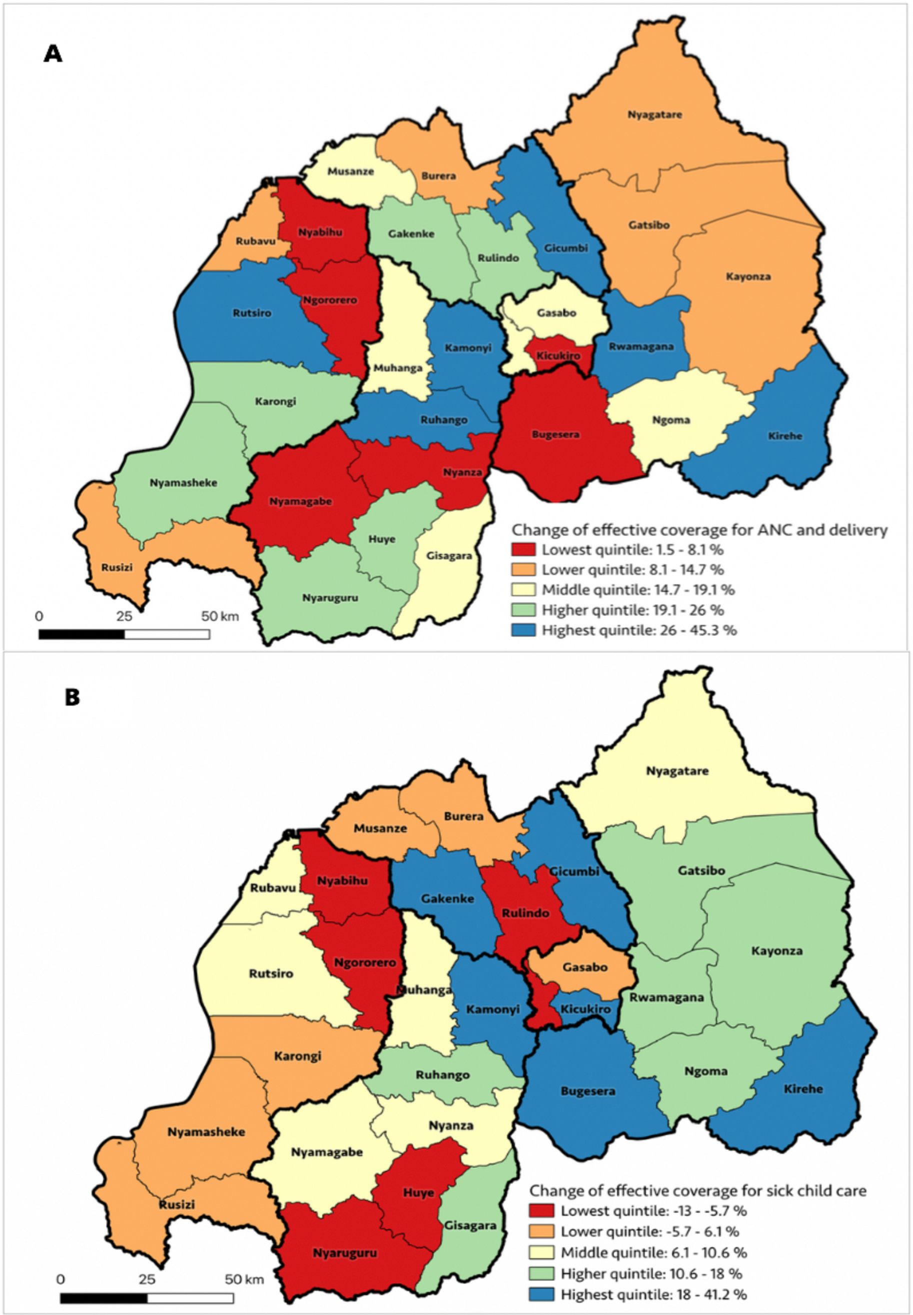

Appendix Figure 7. District level changes in effective coverage of MCH services between 2010 and 2015. (A) Change in effective coverage of maternal health services (effective coverage for ANC and for health facility delivery); (B) Change in effective coverage of child health services (average effective coverage for child diarrhea, pneumonia and fever).

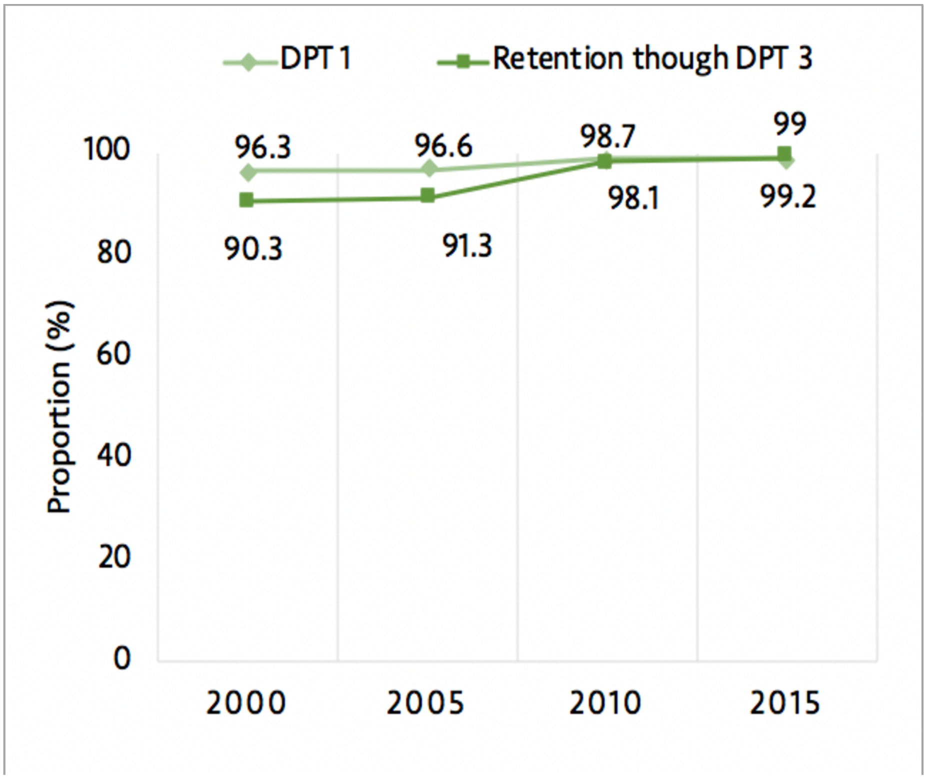

Appendix Figure 8. Coverage and retention of DPT from 2000 to 2015. DPT, diphtheria, pertussis, and tetanus vaccine.
